# Preparation, Characterization, and Activity of Pd/PSS-Modified Membranes in the Low Temperature Dry Reforming of Methane with and without Addition of Extra Steam

**DOI:** 10.3390/membranes11070518

**Published:** 2021-07-09

**Authors:** Cecilia Mateos-Pedrero, Miguel A. Soria, Antonio Guerrero-Ruíz, Inmaculada Rodríguez-Ramos

**Affiliations:** 1 Instituto de Catálisis y Petroleoquímica, CSIC, Calle Marie Curie 2, 28049 Madrid, Spain; cmpedrero@yahoo.es; 2 Departamento de Química Inorgánica y Química Técnica, Facultad de Ciencias UNED, Senda del Rey 9, 28040 Madrid, Spain; aguerrero@ccia.uned.es; 3 UA UNED-ICP (CSIC), Grupo de Diseño y Aplicación de Catalizadores Heterogéneos, 28049 Madrid, Spain

**Keywords:** methane, dry reforming, steam reforming, Pd-membrane, Ru

## Abstract

The external surface of a commercial porous stainless steel (PSS) was modified by either oxidation in air at varying temperatures (600, 700, and 800 °C) or coating with different oxides (SiO_2_, Al_2_O_3_, and ZrO_2_). Among them, PSS-ZrO_2_ appears as the most suitable carrier for the synthesis of the Pd membrane. A composite Pd membrane supported on the PSS-ZrO_2_ substrate was prepared by the electroless plating deposition method. Supported Ru catalysts were first evaluated for the low-temperature methane dry reforming (DRM) reaction in a continuous flow reactor (CR). Ru/ZrO_2_-La_2_O_3_ catalyst was found to be active and stable, so it was used in a membrane reactor (MR), which enhances the methane conversions above the equilibrium values. The influence of adding H_2_O to the feed of DRM was investigated over a Ru/ZrO_2_-La_2_O_3_ catalyst in the MR. Activity results are compared with those measured in a CR. The addition of H_2_O into the feed favors other reactions such as Water-Gas Shift (RWGS) and Steam Reforming (SR), which occur together with DRM, resulting in a dramatic decrease of CO_2_ conversion and CO production, but a marked increase of H_2_ yield.

## 1. Introduction

Pd-based membranes have received much attention owing to their high permeability and selectivity to hydrogen, which is suitable for various applications involving H_2_ separation and purification process [1,2]. Membranes with low thickness are preferable since they improve the hydrogen flow across the membrane and reduce the cost of expensive Pd [3]. Composite Pd membranes, made of a thin Pd layer deposited onto a porous substrate, meet this requirement. The support should have a small external pore size and a smooth surface to form a thin Pd membrane without defects. In this way, PSS are promising supports because of their high-pressure resistance, good mechanical strength, high thermal stability, etc. [4,5,6]. However, a commercial PSS carrier has non-uniform and too large external pore size, which makes more difficult the deposition of a thin dense Pd layer onto this support. Additionally, direct deposition of Pd layer onto PSS surface would give rise to an intermetallic diffusion between the metallic elements of PSS and Pd, decreasing the stability of the membrane.

In this sense, considerable efforts have been made to overcome the interdiffusion problem and decrease both the external pore size and the surface roughness of the PSS substrate. The pretreatment/modification of PSS is extensively described in the literature. Shu et al. [7] coated a layer of TiN by sputtering. Augustine et al. [8] formed an oxide layer by controlled oxidization of porous stainless steel. More common practices are to form an interdiffusion barrier layer by coating a thin layer of a ceramic oxide, among of which CeO_2_ [9], Al_2_O_3_ [10,11], and ZrO_2_ [12,13] are frequently used, although the coating with graphite [14] and zeolites [15] has been also reported.

On the other hand, methane could be valorized by its transformation into syngas, a mixture composed mainly of carbon monoxide and hydrogen, used as a feedstock to value-added products. Such valorization can be carried out by different reactions such as dry reforming, steam reforming, partial oxidation, and auto-thermal reforming. [16]. The dry reforming of methane, DRM, (Equation (1)) is an environmentally friendly process since it helps to the reutilization of CH_4_ and CO_2_, which are two greenhouse effect gases [17,18,19]. The low-temperature DRM yields syngas with a H_2_/CO ratio lower than unity [20,21]. However, combining DRM and SRM (Equation (2); which is generally accompanied by the water-gas shift reaction—Equation (3)) enables to increase of the H_2_/CO ratio, being suitable for the synthesis of valuable liquid fuels via processes such as Fischer–Tropsch or methanol synthesis [22,23]. Furthermore, the presence of steam helps minimize coke formation resulting in a more stable catalyst [21].

(1) $${\mathrm{CH}}_{4(g)}+{\mathrm{CO}}_{2(g)}\;↔\;2{\mathrm{CO}}_{\left(g\right)}+2H_{2(g)}\;\;\;\;\;\Delta H_{r}^{298\;K}=247.0\;\mathrm{kJ}\;{\mathrm{mol}}^{-1}$$

(2) $${\mathrm{CH}}_{4(g)}+H_{2}O_{\left(g\right)}\;↔\;{\mathrm{CO}}_{\left(g\right)}+3H_{2(g)}\;\;\;\;\;\Delta H_{r}^{298\;K}=206.0\;\mathrm{kJ}\;{\mathrm{mol}}^{-1}$$

(3) $${\mathrm{CO}}_{\left(g\right)}+H_{2}O_{\left(g\right)}\;↔\;{\mathrm{CO}}_{2(g)}+H_{2(g)}\;\;\;\;\;\Delta H_{r}^{298\;K\;}=-41.2\;\mathrm{kJ}\;{\mathrm{mol}}^{-1}$$

The application of H_2_-selective membrane reactor technology in reversible reaction as DRM, SRM, and WGS, allows to remove the H_2_ from the reaction zone in order to shift the equilibrium towards the right-side (Le Chatelier’s principle), improving the H_2_ yield. Furthermore, if the DRM is carried out at low temperatures, i.e., in the range 400–500 °C, the process becomes more energy-efficient.

The catalysts more widely used for the reforming process are Ni-based materials due to their good activity and lower costs. However, compared to noble metal-based catalysts (for instance, Rh, Ru, and Ir), they are less active and prone to coke formation. From this point of view, supported Ru catalysts appear very attractive because of their high activity and selectivity as well as stability, in fact, they are much less prone to carbon deposition than the Ni-based catalysts. Previously, we reported the high activity-stability of supported Ru catalysts in the low temperature methane dry and steam reforming reactions [21,24,25]. For application in MRs, the catalyst should be active enough and stable at around 500 °C, since at higher temperatures, the stability of the Pd-membrane can be affected.

Most experimental reports concern the simultaneous dry and steam reforming of methane in a conventional reactor at high-temperature [16,26,27]. Some works related to dry reforming of methane [28,29,30,31,32,33,34,35] or steam reforming of methane in a MR were also reported [36,37]. There are also studies combining steam and dry reforming (biogas reforming) in an MR [38,39,40]. However, in these works, a great amount of steam was used, favoring not only H_2_ production, but also CO_2_ formation (reducing CO_2_ conversion) due to the WGS reaction [17]. On the other hand, little literature is found for methane dry reforming co-feeding small quantities of water at low temperature in an MR. Using a small amount of water enables reducing CO_2_ production and adjusting the syngas ratio (H_2_/CO) to downstream applications.

The objectives of the present work are: (i) to identify the most suitable modified PSS substrate for the fabrication of a dense Pd membrane; in this regard, oxidation or coating, are studied; (ii) to identify the best catalytic formulation among a series of supported Ru samples for their use in the Pd MR; (iii) to study the use of the Pd membrane in the low temperature DRM and to compare it with a CR; and (iv) to analyze the effect of co-feeding a small amount of H_2_O in the activity/selectivity of the selected catalyst in the title reaction.

## 2. Materials and Methods

### 2.1. Synthesis of Catalysts

Ru-containing catalysts were prepared using commercial supports: SiO_2_ (Fluka, Darmstadt, Germany), SiO_2_, (3.5 wt.%) or La_2_O_3_ (7.0 wt.%) stabilized ZrO_2_ (MEL, Manchester, UK). Prior to metal impregnation, supports were calcined in air at 500 °C for 4 h. The surface areas of these materials after calcination were 400, 80, and 105 m^2^/g for SiO_2_, ZrO_2_-SiO_2_, and ZrO_2_-La_2_O_3_, respectively. After calcination, the supports were impregnated with Ru using an aqueous solution of RuCl_3_·H_2_O (Sigma-Aldrich, Darmstadt, Germany) [24]. Afterward, the catalyst precursors were dried in air at 110 °C overnight. In all cases, the Ru loading was close to 4 wt.%. Samples were denoted as Ru/SiO_2_, Ru/ZrO_2_-SiO_2_, and Ru/ZrO_2_-La_2_O_3_ for Ru supported on SiO_2_, ZrO_2_-SiO_2_, and ZrO_2_-La_2_O_3_, respectively.

### 2.2. Synthesis of Membranes

#### 2.2.1. Preparation of PSS Modified Supports

Porous Stainless Steel (PSS) tubes provided by Mott Corporation, Farmington, CT, USA (0.5 μm grade) were employed as support for Pd deposition. The intermetallic diffusion barrier oxides were obtained by either heating the PSS at different temperatures (metallic oxides) or coating the PSS with different ceramic oxides. Before submitting the PSS support to oxidation or coating, it was first cleaned using an ultrasonic bath (at 60 °C) with an alkaline solution (45 g/L NaOH, 65 g/L Na_2_CO_3_, 45 g/L Na_3_PO_4_·12H_2_O, 5 mL/L of an industrial detergent) [41], followed by rinsing with deionized water. Then, the PSS tubes were dipped in isopropanol and dried at 120 °C overnight. Table 1 compiles the modified PSS support herein studied.

Oxidation: PSS supports were oxidized in air at 600, 700, or 800 °C (2 °C/min) for 12 h. Samples were labelled as PSS-600, PSS-700, and PSS-800 for PSS oxidized at 600, 700, and 800 °C, respectively (Table 1).

Coating: A layer of a ceramic oxide (Al_2_O_3_, SiO_2_ or ZrO_2_) was deposited onto the support by the coating technique. Al-isopropoxide (Aldrich, >98%), TEOS (Tetraethyl orthosilicate, Aldrich, 99.0%), and Zr-tetrabutoxide (Aldrich, 80%) were used as starting materials. The coating solution was prepared by the sol-gel method according to the following procedure: an aqueous solution of the given alkoxide was heated at the desired temperature (Table 2). The solution was vigorously stirred for approximately 45 min and HNO_3_ or NH_3_ was then added to the suspension. The resulting solution was stirred for 2 h and used for coating. The molar ratio alkoxide: water and alcohol: acid or base together with the experimental conditions used in this work are gathered in Table 2.

The PSS was placed vertically for a few minutes in a vessel containing the coating sol After coating, the substrate was left to dry at room temperature and successively at 100 °C for 2 h. This was followed by calcination in static air at 500 °C (1 °C/min) for 5 h. Samples were denoted as PSS-Al_2_O_3_, PSS-SiO_2,_ and PSS-ZrO_2_, for PSS coated with Al_2_O_3_, SiO_2_, and ZrO_2_, respectively.

#### 2.2.2. Synthesis of Pd Dense Membranes

Based on characterization results, two of the most suitable modified PSS substrates obtained (cf. Section 2.2.1) were used to prepare Pd dense membranes. These two modified PSS were chosen according to results of He permeation, more uniform surface, absence of cracks, etc. The Electroless Plating technique (EPD) [41] was used to obtain dense Pd layers on the two modified PSS substrates used in this study. The EPD method consisted of the following steps:

Activation: Before Pd deposition, the surface of PSS substrates was seeded with Pd nuclei by the conventional SnCl_2_/PdCl_2_ activation method. The procedure was as follows: the PSS tube was immersed in SnCl_2_ solution (1 g/L) for 5 min. After washing with distilled water, the tube was immersed in PdCl_2_ solution (0.1 g/L) for 5 min, then in HCl (0.01 M) for 2 min, and finally washed with distilled water. This cycle was repeated 6 times.

Plating: Electroless plating was carried out by immersion of the activated PSS tube into a well-stirred plating bath, in an ultrasonic bath kept at 50 °C for 90 min, containing the Pd source, EDTA as the complexing agent, ammonia, and hydrazine as the reducing agent. The conditions and composition of the plating bath were similar to those reported in [42] and are listed in Table 3. The plating technique was repeated until the composite Pd membrane became dense (no He permeance at 25 °C and ΔP = 1 bar). As-prepared Pd membranes were carefully washed with deionized water and then dried at 120 °C overnight. Table 4 shows the Pd composite membranes synthesized.

### 2.3. Characterization

#### 2.3.1. Catalysts

Different physico-chemical characterizations of the catalysts herein utilized, such as H_2_-TPR, CO chemisorption, and XPS, were reported and discussed in a previous work [24].

#### 2.3.2. Membranes

The supports were characterized by Hg porosimetry, X-ray diffraction (XRD), scanning electron microscopy (SEM) combined with Energy dispersive X-ray (EDX), gravimetric analysis, and He permeation measurements.

Hg porosimetry measurements were conducted on a Fisons Pascal 240. Samples of the bare PSS, PSS after oxidation or coating, and Pd-PSS membranes were tested. In each case, a 0.5 cm long specimen was used. The measurements were conducted up to a pressure of 10^9^ Pa.

XRD analysis was performed on a Siemens D500 diffractometer with Cu Kα radiation (1.542 Å). For analysis, a 0.5 cm-long piece was cut from each sample tube while carefully protecting the surface.

Scanning electron microscopy (SEM) analysis was performed on a Philips XL40 microscope equipped with an Energy Dispersion X-ray (EDX) detector. Tubular specimens with a length of about 0.5 cm were first encapsulated with an epoxy resin curing at room temperature. Prior to the SEM analysis, samples were sputtered with a very thin palladium layer to avoid charging.

**Figure 1:**
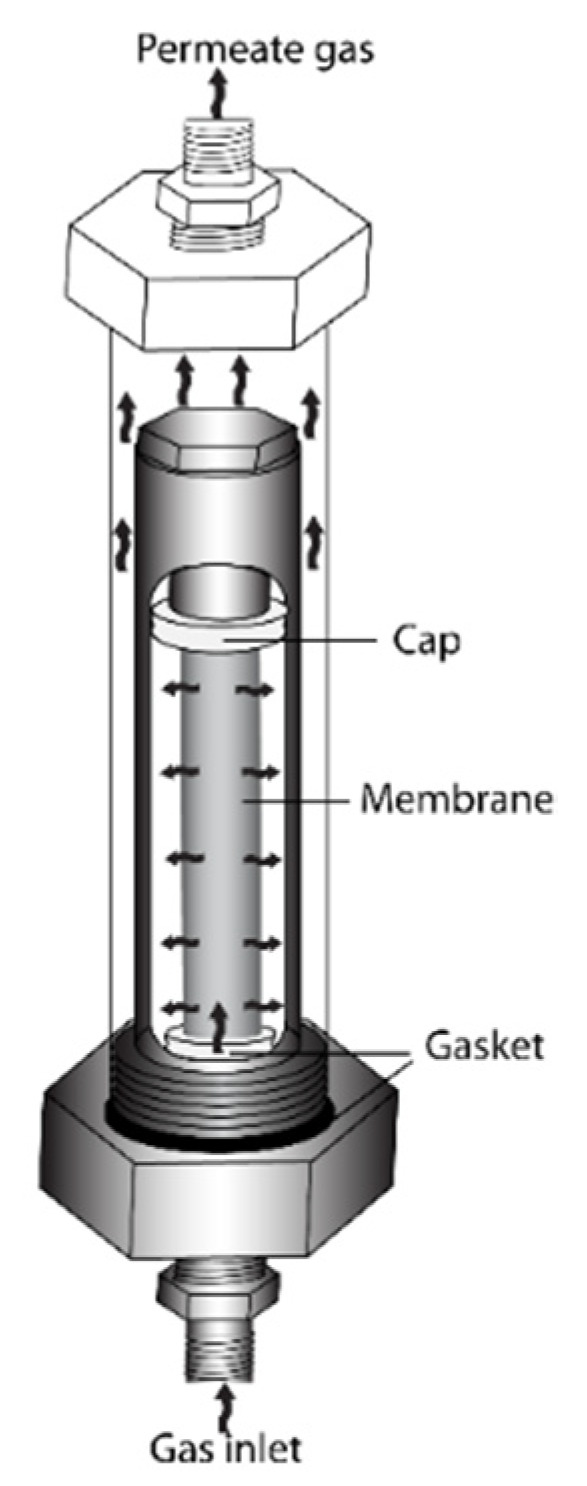
He permeator cell used for the determination of He permeance of the various membranes at room temperature and different transmembrane pressures.

The He permeance of the various tube specimens (5 cm long) was determined at room temperature and at several different transmembrane pressures (0–3.5 atm) before and after each synthesis step by using the home-made He permeator cell shown in Figure 1. The flow of He gas was measured by two bubble flow meters working at two different flow ranges: high flow range (0–10^3^ mL/min) and small flow range (0–5 mL/min).

Hydrogen permeation flux of Pd membrane was measured at 400 and 450 °C. For this study, the same set-up used for catalytic tests was used (more detail will be done below, Section 2.4). The Pd membrane was placed in the middle of the double tubular reactor (inner tube) and sealed with graphite ferrules; first of all, the presence of leaks in the membrane was checked by feeding the reactor with He. No helium permeation through the composite membrane was observed at room temperature, confirming the absence of defects in the palladium film deposited. The membrane was then heated from room temperature to the desired temperature (2 °C/min) under He gas flow to avoid the rupture of the membrane due to H_2_ embrittlement. Then, the Pd membrane was activated with pure H_2_ (at 400 °C, 1 bar, and using 300 mL/min of He as sweep gas), until the permeate H_2_ flow rate was constant. The permeate side was always kept at atmospheric pressure, while on the retentate side, the pressure was varied between 0.5 and 3 bar. At each working condition, the flow rate of hydrogen permeated was measured at the outlet of the permeate side.

A similar procedure was used for the mixture He/H_2_ permeation measurements. However, in this case, a He flow was used as sweep gas at the permeate side in order to maintain throughout the analysis the H_2_ partial pressure lower than that at the retentate side.

### 2.4. Activity Measurements in the Low Temperature Dry Reforming of Methane

#### 2.4.1. Conventional Reactor

Dry reforming tests were performed at atmospheric pressure in a fixed-bed tubular reactor with an inner diameter of 9.5 mm, which was heated in an electric furnace equipped with a PID controller. Fresh 15 mg of fresh catalyst (particle size between 150–250 µm) was mixed with SiC and packed in the middle of the reactor (50 mm bed length). A K-type thermocouple placed in the center of the catalyst bed was used to monitor the temperature.

Prior to the reaction, the catalyst was reduced in situ at 500 °C (maximum operation temperature) for 2 h under 25 vol.% H_2_ in He using a total flow rate of 100 mL/min. After reduction, He was fluxed during 30 min to remove the remaining H_2_ from the reactor. A total flow rate of 100 mL/min (W/F = 2.5 × 10^−6^ g_cat_ h ml^−1^) was used as feed gas mixture, whose volume percent ratio CH_4_:CO_2_:He was 10:10:80. When combining dry and steam reforming, 2 vol.% of water was also fed while maintaining the total flow (CH_4_:CO_2_:H_2_O:He = 10:10:2:78). The gases fed to the reactor were controlled by mass flow controllers (Brook). The composition of water fed was adjusted by controlling the temperature of a thermostatic bath where was placed a gas saturator. The lines before and after the reactor were heated to avoid water condensation. Unconverted water was condensed in a cool trap placed before online gas analysis. The catalytic tests were performed at 400, 450, and 500 °C. More detail about the setup was reported elsewhere [43].

Unconverted reactants and products (CH_4_, H_2_, CO, and CO_2_) were analyzed using a GC (Varian 3400) equipped with teo columns (Porapaq Q and Chromosorb 102) and a TCD. It was verified the absence of both diffusion limitation and catalytic activity of the supports in given conditions. The carbon balance was close to 100% in all cases.

The stability study was carried out under non-equilibrium conditions at 500 °C for 10 h using 15 mg of sample. For the given operating conditions, the composition of the reaction products at the thermodynamic equilibrium was determined using the software ASPEN-HYSYS.

#### 2.4.2. Pd Membrane Reactor

**Figure 2:**
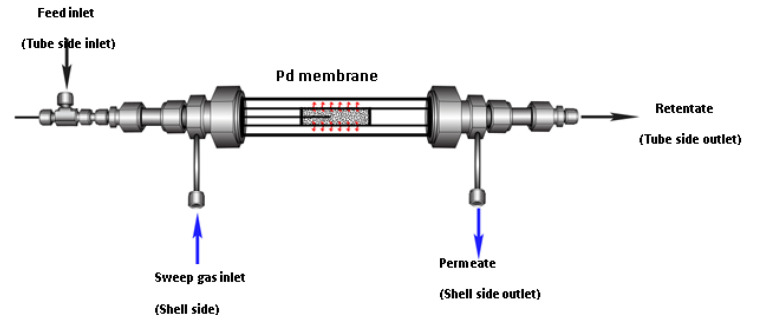
Pd multifunctional membrane reactor (MR) scheme.

The Pd/ZrO_2_-PSS membrane was used for the fabrication of the double tubular MR (Figure 2). The membrane tube was mounted in a stainless steel shell (i.d. 16 mm) and sealed by graphite O-rings at its open end in order to avoid the shell side (permeate side of Pd membrane) stream getting in contact with the internal side (retentate side of the membrane) stream. The catalyst (15 mg), diluted with CSi to avoid hot spots (bed length of 50 mm), was packed in the lumen side. The permeate side of the membrane in all tests was kept at atmospheric pressure. The catalysts were heated in He at 400 °C and then reduced in H_2_ for 2 h. After reduction, the reactor was fed with He and the feed stream gas mixture was then switched to the reactor. The total flow rate, W/F, and feed composition were the same as in the conventional reactor, either for methane dry reforming or bi-reforming. He gas was used as sweep gas.

Inlet and outlet retentate side gases (dry basis) were analyzed by on line GC employing the same equipment as that previously described (Section 2.4.1). During the test, the outlet stream of the permeate side was also analyzed by on-line GC, and hydrogen was the only product detected by the TCD. Additionally, the carbon balance on the retentate side was close to 100%.

For both CR and MR, the conversions of CH_4_ (XCH_4_) and CO_2_ (XCO_2_), the yields of CO (YCO) and H_2_ (YH_2_) were calculated by means of the following equations:

(4) $${\mathrm{XCH}}_{4}\;(\%)=\frac{{{\mathrm{FCH}}_{4}}^{\mathrm{in}}-{{\mathrm{FCH}}_{4}}^{\mathrm{out}}}{{{\mathrm{FCH}}_{4}}^{\mathrm{in}}}\times 100$$

(5) $${\mathrm{XCO}}_{2}\;(\%)=\frac{{{\mathrm{FCO}}_{2}}^{\mathrm{in}}-{{\mathrm{FCO}}_{2}}^{\mathrm{out}}}{{{\mathrm{FCO}}_{2}}^{\mathrm{in}}}\times 100$$

(6) $$\mathrm{YCO}\;(\%)=\frac{{\mathrm{FCO}}^{\mathrm{out}}}{{{\mathrm{FCH}}_{4}}^{\mathrm{in}}}\times 100$$

(7) $${\mathrm{YH}}_{2}\;(\%)=\frac{{{\mathrm{FH}}_{2}}^{\mathrm{out}}}{2\;{{\mathrm{FCH}}_{4}}^{\mathrm{in}}}\times 100$$

where F represents the molar flow (mol min^−1^) of the reagents or products. In the case of the MR, the yield of H_2_ (YH_2_) H_2_^out^ refers to the total H_2_ amount, namely the H_2_ flow measured in both sides, retentate and permeate, of the membrane.

## 3. Results and Discussion

### 3.1. Characterization of Catalysts

The main characterization data for the supported Ru catalysts are summarized in Table 5. More detailed information about characterization of these samples can be found elsewhere [24]. The TPR profile of the fresh sample (a single peak located at 150–161 °C is observed, Table 5) and XPS (BE of Ru3d_5/2_ = 280.4–280.9 eV) of the catalysts after reduction treatment in hydrogen (500 °C for 2 h) confirm the presence of Ru^0^ in the catalysts after reduction (Table 5). Ru/ZrO_2_-SiO_2_ and Ru/ZrO_2_-La_2_O_3_ present comparable dispersion values (28 and −24%) and, hence, similar mean particle sizes (4.7–5.3 nm, respectively), whereas Ru/SiO_2_ shows a lower dispersion (16%) and consequently higher particle size (8.4 nm).

### 3.2. Characterization of PSS Supports and Composite Pd Membranes

#### 3.2.1. Supports

He permeance decreased for all the modified PSS carriers prepared in the present study (Table 1). In the case of oxidized PSS, the higher the oxidation temperature, the lower the He permeance. The decrease in He permeance after oxidation is in the same order of magnitude as that obtained by other authors using similar oxidation treatments [8,42,44,45].

As for the coated PSS, the permeation depends upon the type of oxide used, decreasing as follow: γ-Al_2_O_3_ > ZrO_2_ >> SiO_2_ (Table 1). As the permeation of He decreased after coating indicated that the oxide particles have been effectively introduced into the larger pores of the PSS substrate. This assumption is supported by the decrease in external porosity after coating. In a general way, coating leads to a higher decrease of the He permeance. For instance, Yepes and co-worker [5] stated a reduction of He permeability of 56 and 79% after oxidation in stagnant air and wash coating with Al_2_O_3_, respectively.

Hg porosimetry measurements showed that the maximum pore size (external porosity) significantly decreases after oxidation or coating. It should be noted that the outside porosity reduction is in line with the permeation decrease. In all oxidized PSS, the maximum pore size tends to decrease as the oxidation temperature increases. The oxidation or coating had little effect on the mean pore size (Table 1), indicating that the treatment of PSS did not constrict the internal pore system [42].

**Figure 3:**
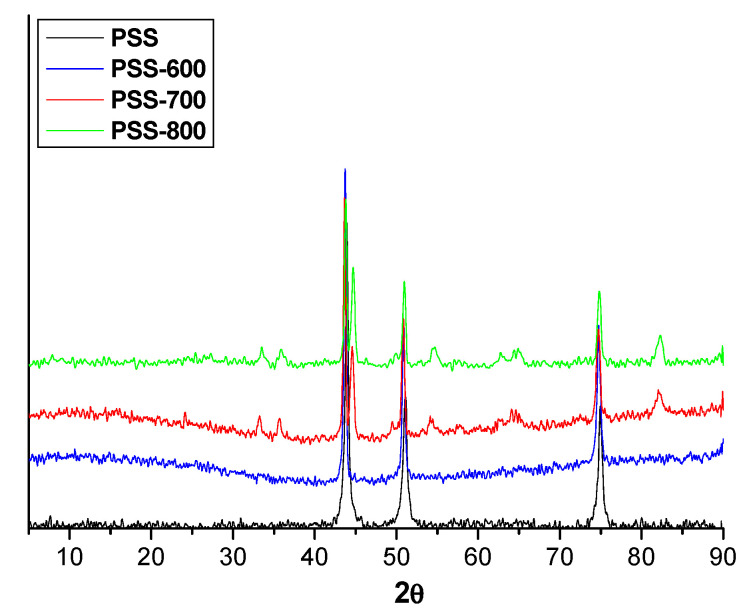
XRD patterns of bare PSS and PSS substrates after oxidation at 600, 700 and 800 °C, respectively.

The sample PSS-600 presents the same XRD pattern as the bare PSS, and only the main peaks of stainless steel at 43.8°, 51.0° and 74.9° are recognized (Figure 3). Nevertheless, for the materials oxidized at 700 and 800 °C, additional features were observed besides the PSS lines, which could be assigned to oxides of Fe, Ni, and Cr. The coated samples did not show appreciable differences in relation to the bare PSS, showing only the characteristic lines of the stainless steel support (data not shown).

**Figure 4:**
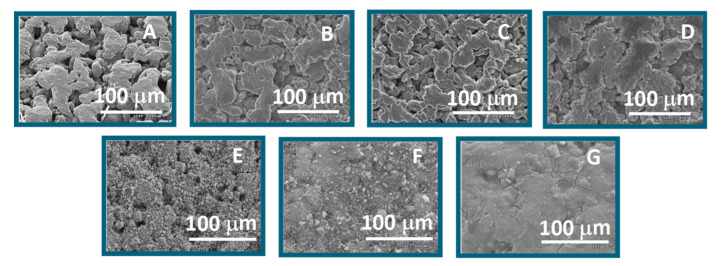
SEM images of: bare PSS (**A**), oxidation at 600 °C (**B**), 700 °C (**C**), and 800 °C (**D**); γ-Al_2_O_3_ (**E**), ZrO_2_ (**F**), and SiO_2_ (**G**) coating.

The surface and cross-sectional morphological characteristics of the modified and bare PSS substrates were examined by SEM (Figure 4). The surface of the modified PSS becomes smoother after oxidation or coating. This effect is even more pronounced for coated samples (especially for ZrO_2_ and SiO_2_ oxides, Figure 4F,G). The surface morphology and roughness of this series of samples depend on the kind of oxide used for coating. For instance, the coating with SiO_2_ leads to the highest surface coverage, although some defects are noticeable in its micrograph (Figure 4G). In the case of the γ-Al_2_O_3,_ some holes are observed (Figure 4E). SEM images strongly suggest that the PSS-ZrO_2_ (Figure 4F) presents a more uniform and homogeneous surface.

The composition on the external surface of several materials was analyzed by EDX. For the oxidized materials, the quantity of Fe on the surface decreases as the oxidation temperature rises from 600 to 700 °C. In contrast, for the PSS support calcined at 800 °C, the Fe/Cr atomic ratio increased dramatically, suggesting the formation of a Fe-rich oxide layer (it is about 6 µm thick) on the outermost layer. Similar observations were obtained elsewhere [42], who claimed the formation of an oxide layer made of an iron-rich layer sitting on top of a mixed Cr and Fe oxide phase.

After coating, the major component on the outer surface of the materials was the corresponding oxide; however, Fe and Cr were still observed, except for PSS-ZrO_2_, which is likely due to the thinness of the oxide layer or the more uniform coverage of PSS. Characterization data for the coated samples indicated that PSS-ZrO_2_ has an intermediate He permeation (decrease of He permeance of c.a. 52% compared with the bare PSS, Table 1), also giving the most uniform and homogeneous surface. On the other hand, the thermal expansion coefficient of ZrO_2_ (1.02 × 10^−5^ K^−1^) is very close to those of PSS (1.73 × 10^−5^ K^−1^) and Pd (1.17 × 10^−5^ K^−1^). All these aspects make ZrO_2_ the most suitable material, among those considered in this work, to be used as an intermetallic diffusion barrier.

Regarding the series of the oxidized carriers, He permeation of PSS-700 ranges between those of PSS-600 and PSS-800 (Table 1). In order to compare both approaches, coating and oxidation, the PSS-700 was also chosen as support for the preparation of Pd membranes. In summary, we have set two substrates, PSS-700 and PSS-ZrO_2_, to prepare Pd membranes, labelled as Pd/ PSS-700 and Pd/PSS-ZrO_2_, respectively.

#### 3.2.2. Pd Membranes

The He flux progressively decreases for the Pd composite membranes as the quantity of plated Pd increases. Pd deposition resulted in a dramatic decrease in porosity, which occurs in the same extent for both Pd/PSS-ZrO_2_ and Pd/PSS-700 membranes.

**Figure 5:**
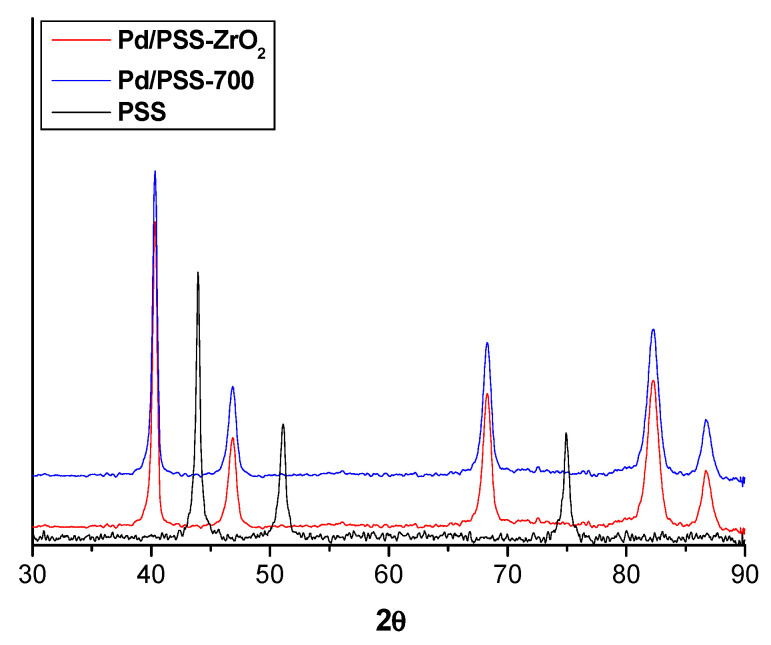
XRD patterns of bare PSS, Pd/PSS-700, and Pd/PSS-ZrO_2_ membranes.

XRD patterns corresponding to Pd/PSS-ZrO_2_ and Pd/PSS-700 membranes only show peaks of metallic Pd (40.1°, 46.7°, 68.1°, and 82.1°), and no peaks attributed to the modified PSS were observed (Figure 5).

**Figure 6:**
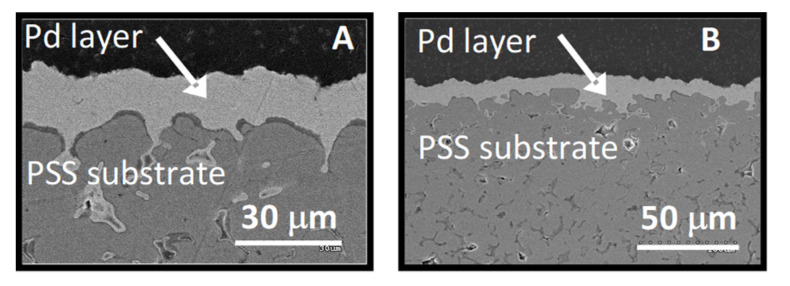
SEM images of the cross section of the oxidized Pd/PSS-700 (**A**) and the coated Pd/PSS-ZrO_2_ (**B**) membranes.

Figure 6 displays the SEM images of the cross-section of both membranes (Pd/PSS-700 and Pd/PSS-ZrO_2_). The average thickness of the Pd layer is close to 17 and 20 µm for Pd/PSS-ZrO_2_ and Pd/PSS-700, respectively. This is in line with the layer thickness estimated by gravimetric analysis for both membranes (Table 4) and suggests that a slightly thinner Pd layer is deposited over the PSS-ZrO_2_ support. It is also noted that a dense Pd film was formed without the presence of fissures. Thus, the leak test validated the absence of detectable defects in both membranes.

It is worth noticing that for the preparation of the dense Pd membrane, the ZrO_2_-coated membrane (Pd/PSS-ZrO_2_) required a lower number of plating cycles, which points to that the coating with ZrO_2_ leads to an increase in the plating effectiveness. To the best of our knowledge, this information has not been published before.

In view of the above results, the Pd/PSS-ZrO_2_ membrane was preferred for carrying out the study of the MR in DRM and the combination of dry and steam reforming of methane. This membrane was characterized in terms of H_2_ permeation, and the results are described in the next section.

#### 3.2.3. Permeation Measurements

The H_2_ permeation through the Pd/PSS-ZrO_2_ membrane was investigated at different transmembrane pressures and temperatures. The H_2_ permeation of the composite Pd/PSS-ZrO_2_ membrane at 400 and 450 °C against the transmembrane pressure (it is the difference between the pressure in the feed and in the permeate side, ∆P = P_feed_ − P_permeate_) showed that the hydrogen permeation rate is directly proportional to the difference in the square root of the feed and permeate pressures (data not shown). This is consistent with the Sieverts’ law [46] and indicates that the rate-determining step for hydrogen permeation is the hydrogen diffusion through the dense Pd layer [47].

**Figure 7:**
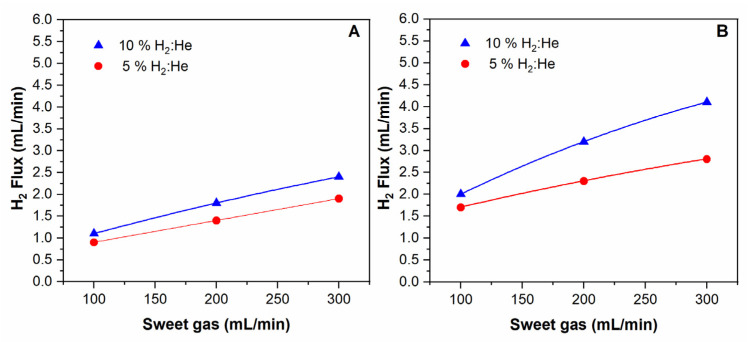
H_2_ permeated flux rate of the composite membrane as a function of the sweep gas flow for mixtures H_2_/He at: 400 °C (**A**) and 450 °C (**B**).

The hydrogen permeation rate as a function of the sweep gas flow was measured for binary mixtures of hydrogen/helium. The results are shown in Figure 7. It can be observed that the hydrogen permeation rate increased with the sweep gas flow rate. The increase of the sweep gas flow rate reduces the hydrogen partial pressure in the permeate side, producing an increase in the H_2_ flow rate through the membrane (higher driving force); as a result, the hydrogen permeation flux increases.

### 3.3. Activity in the Low-Temperature DRM

#### 3.3.1. Activity Measurements Performed in the Conventional Reactor

**Figure 8:**
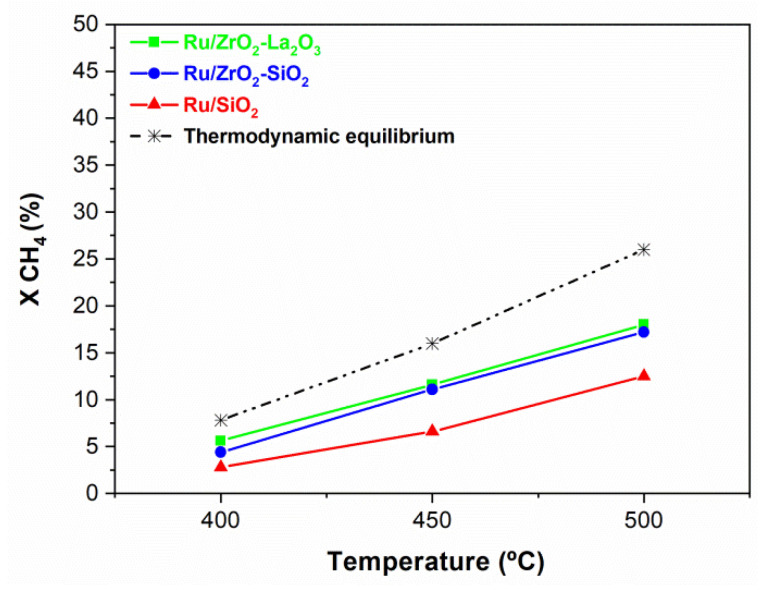
Methane conversion as a function of the temperature for the DRM reaction carried out in the CR over Ru/ZrO_2_-La_2_O_3_ (■), Ru/ZrO_2_-SiO_2_ (●), and Ru/SiO_2_ (▲); Feed composition: CH_4_: CO_2_; He = 10:10:80; Total flow rate = 100 mL/min: P = 1 bar. Dotted line shows the thermodynamic equilibrium.

Figure 8 shows the reforming activities obtained in the fixed bed conventional reactor (CR) for the Ru-based catalysts in terms of methane conversion (%) in the DRM reaction as a function of the temperature (from 400 to 500 °C; this temperature range has been explored because it is the temperature interval applicable in the MR). Equilibrium methane conversion predicted by thermodynamics is also included in this figure for comparison. As can be seen under the selected experimental conditions, all the supported Ru catalysts show conversion values below thermodynamic equilibrium, and their activity increases with the reaction temperature. Comparing the three samples, it is observed that, at a given temperature, the activity of both Ru/ZrO_2_-La_2_O_3_ and Ru/ZrO_2_-SiO_2_ is nearly the same and greatly higher than that of Ru/SiO_2_, particularly at high temperatures; for instance at 500 °C, the conversion of methane is 18.0, 17.2, and 12.5% for Ru/ZrO_2_-La_2_O_3_, Ru/ZrO_2_-SiO_2,_ and Ru/SiO_2_, respectively.

**Figure 9:**
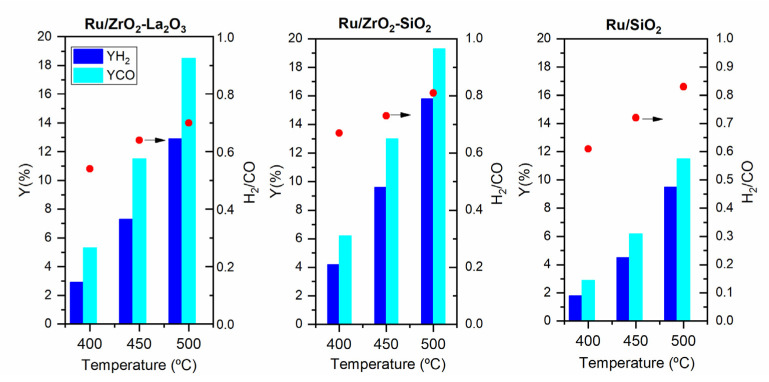
Yield of H_2_ and CO, and H_2_/CO ratio as a function of the temperature for the DRM reaction carried out in the CR over Ru/ZrO_2_-La_2_O_3_, Ru/ZrO_2_-SiO_2_, and Ru/SiO_2_; Feed composition: CH_4_: CO_2_: He = 10:10:80; Total flow rate = 100 mL/min: P = 1 bar.

Figure 9 exhibits the yield of H_2_ (YH_2_) and CO (YCO) obtained over the three prepared catalysts at various reaction temperatures. The H_2_ to CO molar ratio is also reported in this figure. Similar to the increased conversions, the H_2_/CO ratio also increased with reaction temperature herein analyzed, but it is always smaller than the stoichiometric value of 1 for the DRM reaction (Equation (1), demonstrating the occurrence of the reverse of water-gas shift (RWGS) reaction (H_2_ + CO_2_ ↔ H_2_O + CO) as well. This side reaction consumes H_2_ and produces CO resulting in the decrease of the H_2_ to CO ratio. For the same reason, regardless of the catalyst and the temperature, the CO_2_ conversion is always higher than the CH_4_ conversion (data not shown).

##### Stability Measurements Performed in the CR

**Figure 10:**
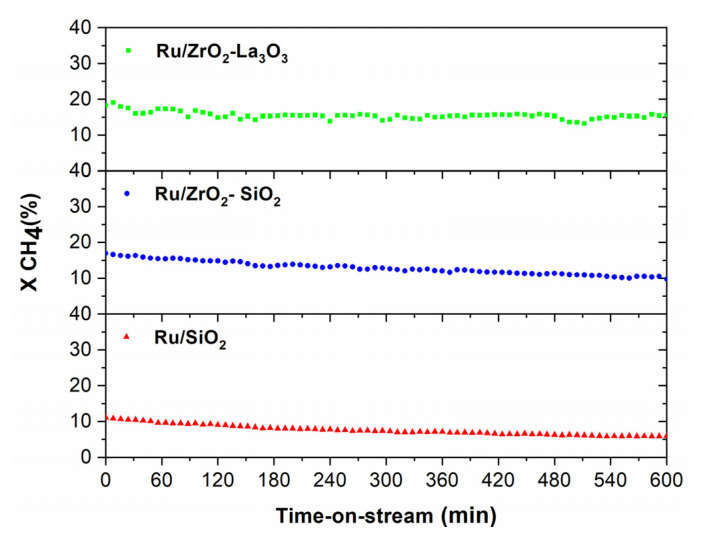
Methane conversion versus time-on-stream during DRM reaction carried out in the CR at 500 °C over supported Ru catalysts; Ru/ZrO_2_-La_2_O_3_ (■), Ru/ZrO_2_-SiO_2_ (●), and Ru/SiO_2_ (▲); Feed composition: CH_4_: CO_2_: He = 10:10:80; Total flow rate = 100 mL/min: P = 1 bar.

The stability of the catalysts is a crucial aspect to be considered for their application in an MR. With this aim, some tests were conducted at 500 °C for 10 h over the Ru-containing catalysts, and the results are displayed in Figure 10. The initial conversion for the Ru/ZrO_2_-La_2_O_3_ and Ru/ZrO_2_-SiO_2_ catalysts was 18.0 and 17.2%, respectively, and decreased during the 10 h of reaction to 15.3 and 9.7% of CH_4_ conversion, respectively. The Ru/SiO_2_ catalyst exhibited a significantly lower initial conversion (12.5%) and, likewise to the others catalysts, showed deactivation with time on stream (5.9%). As illustrated in Figure 10, the degree of deactivation for Ru/ZrO_2_-SiO_2_ and Ru/SiO_2_ catalysts (43 and 53%, respectively) was greatly higher than for Ru/ZrO_2_-La_2_O_3_ (15%). Since Ru/ZrO_2_-La_2_O_3_ is the most performing and stable catalyst amongst all the catalysts considered in this work, it was selected for the study carried out in both CR and MR in the following sections.

##### Influence of Co-Feeding Water in the CR

**Figure 11:**
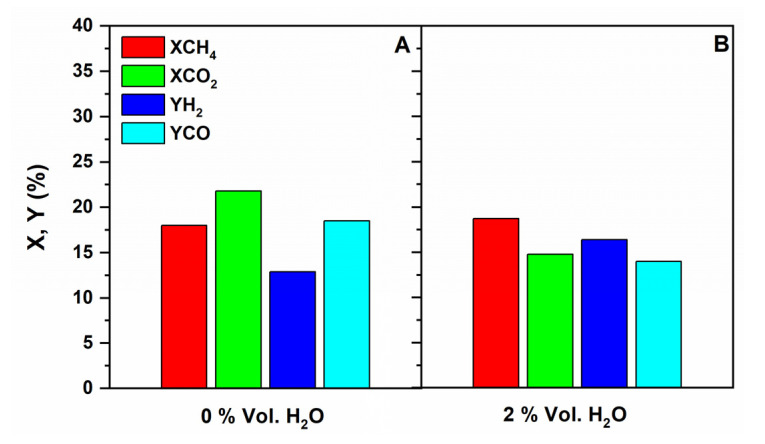
Conversions and yields for the DRM reaction carried out in the CR over the Ru/ZrO_2_-La_2_O_3_ catalyst with (**A**) and without H_2_O (**B**) in the feed; Feed composition: CH_4_: CO_2_: He = 10:10:80 (**A**) and CH_4_:CO_2_:H_2_O:He = 10:10:2:78 (**B**); Total flow rate = 100 mL/min; T = 500 °C; P = 1 bar.

According to other published works, the addition of H_2_O to the feed of DRM inhibits carbon formation (more stability), which is favored in the dry reforming operating conditions, and might produce syngas with desired compositions (H_2_/CO ratio) [23,48]. Figure 11 compares CH_4_ and CO_2_ conversions and H_2_ and CO yields in the low-temperature DRM with and without co-feeding H_2_O (2 vol.%) in the CR. As it can be seen in this figure, the addition of H_2_O into the feed led to the following effects: (i) significant decrease in CO_2_ conversion; (ii) no influence on CH_4_ conversion, which remained mainly unchanged: and (iii) increase in H_2_ yield and a marked decrease in the yield of CO, as a result, the H_2_/CO ratio increases (from 0.7 to 1.2).

The fact that methane conversion almost does not change, suggests that the SRM (Equation (2)) reaction did not take place to any appreciable extent when co-feeding small amounts of water. Our results are reasonably understood when considering the involvement of WGS reaction (Equation (3)), which would account for the higher H_2_/CO ratios (>1) and at the same time, for the decreasing conversion of CO_2_ (some CO_2_ is being produced by the WGS reaction).

#### 3.3.2. Tests Performed in the Multifunctional Pd MR

Two kinds of tests were performed in the MR: in the absence and in the presence of a small amount of H_2_O (2 vol.%) into the feed. Next sections describe the effect of co-feeding steam on the activity of Ru/ZrO_2_-La_2_O_3_ catalyst when using the MR.

##### The Effect of the Pd Membrane: CR vs. MR

(a) Without steam in the feed

**Figure 12:**
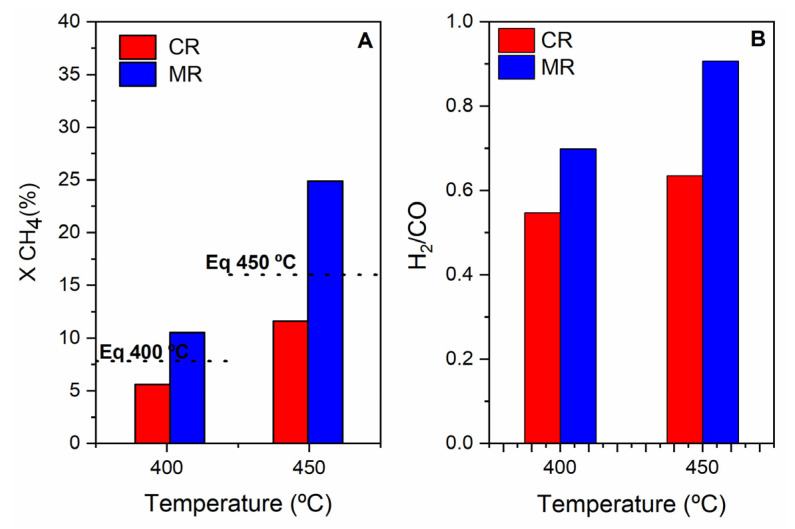
Methane conversion (**A**) and H_2_/CO ratio (**B**) for the DRM reaction in the MR and CR over Ru/ZrO_2_-La_2_O_3_ catalyst at 400 and 450 °C.; Feed composition: CH_4_:CO_2_:He = 10:10:80; Total flow rate = 100 mL/min: P = 1 bar. Sweep Gas: 300 mL/min (MR). The dotted line represents the thermodynamic CH_4_ conversion value at a given temperature.

The CH_4_ conversion and H_2_ to CO molar ratio over the Ru/ZrO_2_-La_2_O_3_ catalyst in the MR at 400 and 450 °C are presented in Figure 12. For comparison purpose, the results obtained in the CR and the thermodynamic values of CH_4_ conversion are also included. The CH_4_ conversion for the CR is lower than for the MR; for example, at 450 °C, the CH_4_ conversions are 11.6 and 24.9%, respectively. This figure also shows that the use of MR allows achieving conversions above the thermodynamic equilibrium. This effect is particularly noticeable at 450 °C (CH_4_ conversion corresponds to 39% improvement from the equilibrium value) because the hydrogen flux through the membrane increases when both the temperature and hydrogen partial pressure in the reaction side increase. It is interesting to note that, H_2_/CO ratio in the MR is always higher compared with the value obtained in the CR (Figure 12B). This suggests that the reverse of the WGS reaction is hindered by the membrane, which removes H_2_, increasing the H_2_/CO ratio (it approaches the stoichiometric value of one). Other authors observed similar behavior when studying the effect of a hydrogen perm-selective membrane on the methane dry reforming process [28,49,50].

It is known that as the transmembrane pressure (difference between retentate and permeate side) increases, so does the H_2_ permeation flux due to the increase of the hydrogen driving force. However, higher pressure leads to decreased CH_4_ conversion, following Le Chatelier’s principle, since there is an increase in the number of moles (Equation (1)) [19,51]. In this sense, Oyama et al. [19] reported that, in a membrane reactor, both CH_4_ and CO_2_ conversion continuously decrease with the pressure, approaching these obtained in the conventional reactor. Thus, in this work, the effect of the pressure in the retentate side on the activity was not studied; but, the hydrogen partial pressure in the permeate side was minimized using a sweep gas, increasing the permeation driving force across the membrane.

(b) With steam in the feed

**Figure 13:**
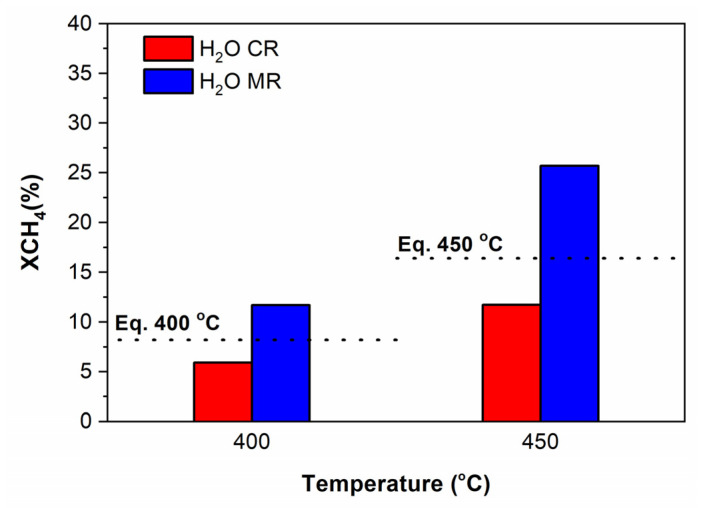
Effect of co-feeding H_2_O (2 vol.%) on the activity over Ru/ZrO_2_-La_2_O_3_ catalyst at 400 and 450 °C in the MR and a CR; Feed composition: CH_4_:CO_2_:H_2_O:He = 10:10:2:78; Total flow rate = 100 mL/min: P = 1 bar. Sweep Gas: 300 mL/min (MR). The dotted line represents the thermodynamic CH_4_ conversion value at a given temperature.

**Figure 14:**
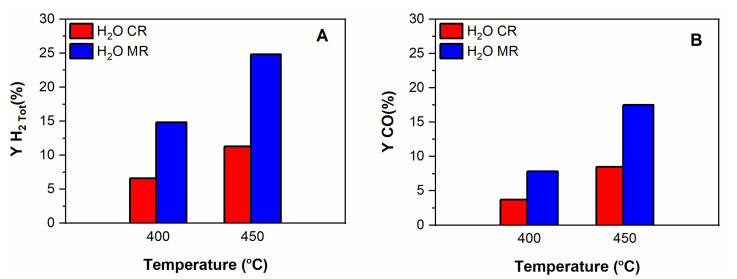
Effect of co-feeding H_2_O (2 vol.%) on the yields of H_2_ (**A**) and CO (**B**) over Ru/ZrO_2_-La_2_O_3_ catalyst at 400 and 450 °C in the MR and CR.; Feed composition: CH_4_:CO_2_:H_2_O:He = 10:10:2:78; Total flow rate = 100 mL/min: P = 1 bar. Sweep Gas: 300 mL/min (MR).

The performances of Ru/ZrO_2_-La_2_O_3_ catalyst in both CR and MRs are shown in Figure 13 and Figure 14. The thermodynamic equilibrium CH_4_ conversion at 400 and 450 °C is also included in Figure 13. Comparison of data shown in Figure 13 and Figure 14 indicates that the use of the MR leads to: (i) greatly enhancing CH_4_ conversion, (ii) a significant improvement of H_2_ and CO yields.

The remarkable increase in CH_4_ conversion is explained as the shift of those reactions in which methane is a reaction reagent and hydrogen is a reaction product (mainly DRM), since the latter is continuously removed from the system by the Pd membrane. On the other hand, the membrane causes the WGS reaction shift towards right-side even more, resulting in an increase of H_2_/CO ratio compared to the CR. This is important to boost H_2_/CO ratio in DRM, in fact and although the stoichiometric value is one, the combination of a MR and the addition of a small amount of steam to DRM contributes to greatly enhancing the H_2_/CO ratio (close to 1.89 and 1.41 at 400 and 450 °C, respectively).

The effect of co-feeding H_2_O on the catalytic performance of Ru/ZrO_2_-La_2_O_3_ in the MR at 450 °C is shown in Table 6. The conversion of CO_2_ considerably decreases by co-feeding H_2_O. In addition, contrary to CO_2_, little effect of H_2_O is observed in the conversion of CH_4_. Significant suppression of CO and a huge enhancement of H_2_ production in the presence of H_2_O is observed (Table 6): as a result, H_2_/CO ratio greatly increases. This behavior is easily understood considering the participation of steam reforming (SR) and mainly water gas shift (WGS). The H_2_O co-feeding would cause the shift of the SR (Equation (2)) and WGS (Equation (3)) equilibrium to the right side, resulting in the diminution of CO_2_ conversion and CO yield while increasing CH_4_ conversion and H_2_ production. The minor effect of H_2_O on the CH_4_ conversion could be because, in our operating conditions, the participation of SR is negligible.

Although this effect was already noticed in the CR, it occurs to a much *greater* extent in the MR since the continuous H_2_ removal by the Pd membrane causes WGS equilibrium shifts towards the right side, increasing H_2_ production while decreasing CO yield. Thus, the combination of both MR and H_2_O co-feeding (a very small amount) allows obtaining a H_2_/CO ratio higher than the stoichiometric value (1). The significance of this observation for the DRM process is great. Simply by combining MR and adjusting the steam-to-methane feed ratio, the H_2_ to CO ratio can be adjusted to the needs of the downstream process avoiding additional units.

## 4. Conclusions

The modification of PSS by coating results in a better substrate for Pd deposition by Electroless Plating technique (EPD). Thus, the ZrO_2_-coated PSS allows a more effective plating process as well as the deposition of a thinner Pd layer. A thin dense Pd membrane was developed by depositing Pd by EPD onto a ZrO_2_-coated PSS support. From the study of several supported Ru catalysts in a CR for the low-temperature DRM, the Ru/ZrO_2_-La_2_O_3_ was identified as the best catalytic formulation in terms of activity, selectivity, and stability. The effect of H_2_O as gaseous dope has also been evaluated. It has been demonstrated that the addition of a small amount of H_2_O contributes to enhancing H_2_ yield and consequently the H_2_-to-CO ratio by favoring the WGS reaction.

Comparison of the experimental results obtained in the MR with those obtained in the CR, reveals the beneficial effects of using the former on the activity and H_2_ yield of a Ru/ZrO_2_-La_2_O_3_ catalyst in the studied reaction. When no water is fed, although the Pd-membrane favors the increase of the H_2_ yield, the ratio H_2_-to-CO is always lower than one. However, when a small amount of water is fed, it is possible to obtain H_2_-to-CO ratio higher than one.

Further improvement of the H_2_ yield in the methane dry reforming would be obtained if the hydrogen flux across the membrane is increased. In this sense, as further works, the hydrogen permeability could be enhanced by using binary Pd alloy (e.g., Pd-Ag) membranes, which can improve the membrane stability at lower temperatures and favor the hydrogen permeability, shifting, even more, the equilibrium reaction. 

## Figures and Tables

**Table 1:** Modified-PSS support assessed.

Sample	Modification of PSS Support	^a^ Dp (µm)	ΔHe Permeation ^b^ (%)
PSS-600	Oxidation at 600 °C for 12 h	4.4	−17
PSS-700	Oxidation at 700 °C for 12 h	4.5	−54
PSS-800	Oxidation at 800 °C for 12 h	4.6	−76
PSS-SiO_2_	Coating with SiO_2_	4.6	−68
PSS-Al_2_O_3_	Coating with Al_2_O_3_	4.8	−23
PSS-ZrO_2_	Coating with ZrO_2_	4.6	−52

**^a^** D_p_, mean pore diameter derived from Hg porosimetry. D_p_ for the bare PSS is 4.4 µm. **^b^** ΔHe is the He flux through the support after oxidation or coating expressed as the percentage of the original He flux at 25 °C and ΔP = 1 bar.

**Table 2:** Materials and experimental conditions used for coating solution synthesis.

Component	Silica Coating	Alumina Coating	Zirconia Coating
Alkoxide	TEOS	Al-isopropoxide	Zr-tetrabutoxide
Peptizing agent	NH_3_	HNO_3_	HNO_3_
Solvent	Ethanol	Isopropanol	Butanol
* Alkoxide: H_2_O	0.25:9	1.2:105	0.04:8
* Alcohol: Acid or base	5.5:0.45	6:0.1	0.25:0.02
Temperature (°C)	50	80	60

*: molar ratio.

**Table 3:** Composition of the electroless plating bath for Pd and plating conditions.

Component	Pd Bath
Pd(NH_3_)_4_Cl_2_·H_2_O	4 g/L
NH_4_OH (28%)	198 mL/L
Na_2_EDTA.2H_2_O	40 g/L
N_2_H_4_ (1 M)	5.6 mL/L
pH	10–11
Temperature	50 °C

**Table 4:** Pd composite membranes assessed.

Sample	He Permeation * (m^3^/m^2^ h)	Modification of PSS Support	Pd Thickness Gravimetric (μm)
Pd/PSS-ZrO_2_	dense	Coating with ZrO_2_	17
Pd/PSS-700	dense	Oxidation-700 °C; 12 h	20

*: He flux at 25 °C and ∆P = 1 bar.

**Table 5:** TPR data (temperature of the maximum peak of the TPR profile); CO microcalorimetry (adsorbed amounts: N_ads_; dispersions: D; mean Ru particle sizes: d) and XPS data (Ru3d_5/2_ Binding Energy and XPS atomic ratios) for the supported Ru catalysts.

Catalyst	TPR (°C)	N_ads_ (μmol/g_cat_)	D (%)	d (nm)	* B. E. (eV)	* XPS at. Ratio (%)	
					Ru3d_5/2_	Ru/Si	Ru/Zr
Ru/SiO_2_	150	62	16	8.4	280.4	0.009	—
Ru/ZrO_2_-SiO_2_	159	112	28	4.7	280.9	—	0.05
Ru/ZrO_2_-La_2_O_3_	161	85	24	5.3	280.6	—	0.08

*: XPS data correspond to catalysts after reduction in H_2_ at 500 °C for 2 h.

**Table 6:** Conversions and yields for the Ru/ZrO_2_-La_2_O_3_ catalyst measured in the activity essays of the DRM carried out in a Pd MR with and without H_2_O in the feed at 450 °C.

H_2_O Vol. % in the Feed	XCH_4_	XCO_2_	YH_2_	YCO	H_2_/CO Ratio
0	24.9	25.2	19.4	21.4	0.91
2	25.7	18.5	24.8	17.5	1.41
